# Potential Usefulness of Tracking Head Movement via a Wearable Device for Equilibrium Function Testing at Home

**DOI:** 10.1007/s10916-022-01874-4

**Published:** 2022-10-11

**Authors:** Yoshiharu Yamanobe, Masato Fujioka, Masanao Ohashi, Hiroyuki Ozawa

**Affiliations:** 1 Department of Otolaryngology-Head and Neck Surgery, Keio University School of Medicine, Tokyo, Japan; 2 Department of Molecular Genetics, Kitasato University School of Medicine, Kanagawa, Japan; 3 Clinical and Translational Research Center, Keio University Hospital, Tokyo, Japan; 4 Department of Otolaryngology-Head and Neck Surgery, Kitasato University School of Medicine, Kanagawa, Japan

**Keywords:** Head movement, Postural sway, Wearable accelerometer sensor, Stabilometry, Digital healthcare

## Abstract

Many studies have reported the use of wearable devices to acquire biological data for the diagnosis and treatment of various diseases. Balance dysfunction, however, is difficult to evaluate in real time because the equilibrium function is conventionally examined using a stabilometer installed on the ground. Here, we used a wearable accelerometer that measures head motion to evaluate balance and examined whether it performs comparably to a conventional stabilometer. We constructed a simplified physical head-feet model that simultaneously records “head” motion measured using an attached wearable accelerometer and center-of-gravity motion at the “feet”, which is measured using an attached stabilometer. Total trajectory length (r = 0.818, p -false discovery rate [FDR] = 0.004) and outer peripheral area (r = 0.691, p -FDR = 0.026) values measured using the wearable device and stabilometer were significantly positively correlated. Root mean square area values were not significantly correlated with wearable device stabilometry but were comparable. These results indicate that wearable, widely available, non-medical devices may be used to assess balance outside the hospital setting, and new approaches for testing balance function should be considered.

## Introduction

In recent years, small sensors that can be attached to the body to acquire biological data, called “wearable devices,” have been used extensively worldwide [1]. Wearable devices are utilized in clinical research as well as in Human–Computer Interaction (HCI) areas in many medical and healthcare fields including neurology [2], cardiology [3], respiratory medicine [4], and rehabilitation medicine [5]. In the field of neurology, for example, applications that collect data from accelerometers in mobile devices have been used to quantify postural instability in Parkinson's disease [6]. Further, postural sway in concussion patients has been assessed using both stabilometry and wearable devices [7]. In the cardiovascular field, many patients who were notified by smartwatches of possible arrhythmias and underwent electrocardiography showed premature ventricular contraction (PVC) and other cardiac diseases, suggesting prior cardiovascular high risk [8]. Much research has been conducted using measurements obtained from wearable devices used in clinical practice. For instance, some researchers have studied the coordination of head and trunk movement using wearable devices in patients with surgically induced unilateral vestibular dysfunction to describe daily changes in their balance disorder [9]. The trial was projected after a decade of straggling by several different groups of digital head-posture measurement systems to track head motion [10]. Conversely, stabilometry has become the most common way to test balance function and has been approved by agencies in modern countries to track changes in lateral center of pressure (COP). Studies have used stabilometry to assess patients with neurological disorders, for example, multiple sclerosis and atrial vertigo [11]. Despite these attempts in preclinical/clinical trials, few studies have compared the parameters of wearable device facilitated head motion tests with those of balance function or stabilometry.

In this study, we constructed a simplified model to compare movements of head motion and center of pressure at the stabilometer. To simplify measurements, we built a system in which a wearable accelerometer (“head” part) and a plate of the stabilometry (“foot” part) move as one unit because they are fixed on a tripod. “Head” accelerometer motion and “foot” center-of-gravity motion were simultaneously measured, and the sets of motion data were compared via cross-correlation analysis. The total length of the trajectory drawn by “head” motion and the area calculated using the trajectory were investigated, which revealed an association between head and center-of-gravity sway. We believe small and low-cost wearable devices that can be attached to the head may provide extensive information regarding balance and equilibrium. Daily monitoring has the potential to provide important time-related data, which are indispensable for understanding changes in unstable symptoms that cannot be measured using existing large medical devices, such as stabilometers. Additionally, research acquiring and analyzing biometric data with small devices can connect with other biometric data and develop HCI in medicine. For example, the vestibulo-ocular reflex is an EOG signal that has not yet been used in other wearable applications. If methods evaluated in this study were developed and combined with other wearable data-acquisition devices, data acquisition outside the hospital has the potential to provide disease diagnosis with quality comparable to that of medical testing [12].

## Methods

### Devices

#### Reference inertial measurement device

**Fig. 1:**
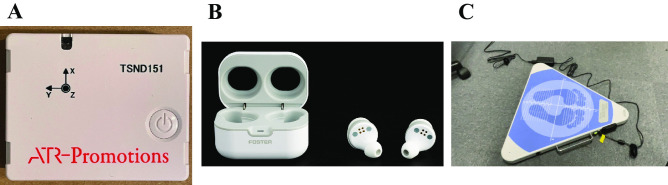
Devices used in the experiment. **A** IMU (Inertial Measurement Unit). **B** RN002 TW (Wearable devices with accelerometer). **C** Stabilometer (stabilometric recordings)

The inertial measurement unit (IMU) detects inertial motion with high precision using a composite of a 3-axis gyro sensor that measures rotation and changes in direction, and a 3-axis acceleration sensor that measures changes in axial velocity. The IMUs used as reference inertial measurement devices in this study were compact wireless multifunction sensors (TSND151, ATR-Promotions, Kyoto, Japan) (Fig. 1A). The size of the IMU was 40 × 50 × 14 mm and its weight was approximately 27 g. The IMU consists of a triaxial accelerometer (InvenSense MPU-9250, TDK, Tokyo, Japan), triaxial gyroscope (AMI306, AICHI STEEL, Aichi, Japan), and triaxial magnetometer (MPL3115A2, NXP Semiconductors N.V., Eindhoven, Netherlands). IMU data were recorded at 100 Hz using Bluetooth, given that the ISPGR Standardization Committee recommends measuring the Vibration and Sway Density Parameters in the Stabilometer at 100 Hz [13] and we followed the policy of the commission and employed a sampling frequency for all of the instruments to match measurement conditions.

#### Wearable devices with an accelerometer (RN002 TW)

In this study, a wearable RN002 TW accelerometer (Foster Electric Company, Limited, Tokyo, Japan) was used (Fig. 1B). This device includes earbuds and is equipped with an accelerometer and angular rate (InvenSense ICM-42605, TDK, Tokyo, Japan) and magnetic sensors (AK09918C, Asahi Kasei Microdevices Corporation, Tokyo, Japan). Using this device, acceleration was recorded at 100 Hz via Bluetooth.

#### Stabilometry

We used the Gravicorder GW-5000 (Anima Co. Ltd., Japan) for stabilometric recordings (Fig. 1C). The device contained vertical force transducers that were used to measure instantaneous fluctuations in COP values. Outcome measures included total length of COP sway and the root mean square of the area traced by COP sway. Measured data were recorded at 100 Hz.

### Head motion measurement data confirmation via stabilometery

**Fig. 2:**
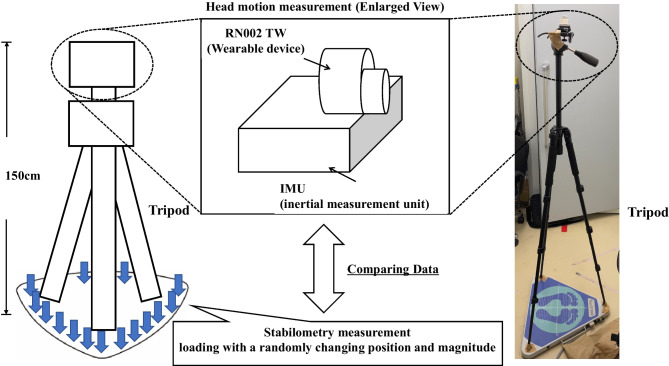
Experiment of head motion measurement data confirmation via stabilometery setup. The IMU and RW002 TW were fixed and attached to the head of the tripod. Data recording continued for 60 s, and a load with a randomly changing position and magnitude was applied to the stabilometer to compare head measurement and stabilometry measurement

Measurements of head and center of gravity sway are shown in Fig. 2. The head motion measurement device was calibrated by fixing it to a tripod such that the XY axis of each device was aligned with the XY axis of the stabilometer. The tripod was placed and fixed to the stabilometer such that its center of gravity was zero. The height of the tripod (distance between the stabilometry and IMU) was 150 cm, to reproduce the natural position of a human head.

When the head motion measurement device and stabilometer started recording the data, force was applied to the stabilometer for a short duration, which functioned as a signal that facilitated the synchronization of analyzed motion. Subsequently, the force was released and the stabilometer returned to the neutral position. Subsequently, a load of arbitrary magnitude was applied at appropriate time intervals in a vertical direction to any part of the periphery of the stabilometry plate that did not interfere with the legs of the tripod legs. In this manner, we were careful to randomize the location, magnitude, and timing of the load to obtain measurements via IMU, RN002 TW, and stabilometry. Data recording continued for 60 s, and a load with a randomly changing position and magnitude was applied to the stabilometer. RN002 TW-recorded data were analyzed 12 times, three times each for all four individuals. One data point was excluded due to recording errors.

### Data processing

The following data were analyzed: triaxial acceleration measured via head motion measurement devices after the trigger signal (IMU, RN002 TW) and biaxial COP displacement trajectory measured via stabilometry.

### Axis selection

We matched XY axis data obtained via stabilometry with triaxial acceleration data measured using IMU and RN002 TW. Z-axes (vertical axis) of stabilometry data for each device were excluded from the study because they were much smaller than XY axis data.

#### Calculating trajectory using a double integral

In general, acceleration sensors also detect DC (Direct Current) components that do not change over time. Even if no acceleration was applied, the output does not become zero, and a zero offset may be output. Therefore, the average value of all measured data (from after the trigger signal to the end of load application) was subtracted.

The zero-offset component is caused by changes in the ambient environment's temperature and the sensor's characteristics. Its value is usually almost constant, unless the measurement is made over an extended period. In the case of HPFs (High pass filters), the cutoff frequency, type, transition band characteristics, stopband characteristics, and passband characteristics must all be appropriately selected, and the HPFs must be designed. However, since the objective here was to remove the zero-offset component, which can be assumed to remain unchanged over time during the measurement, we adopted the method of removing the mean value as it is the simplest method that requires no filter design. Velocity data were estimated by integrating the acceleration data after average acceleration data subtraction was performed using data obtained from the IMU and RN002 TW. Similarly, for obtained velocity data, displacement data were estimated by integrating velocity data after subtracting the average value of all calculated velocity data.

#### Data confirmation via cross-correlation

**Fig. 3:**
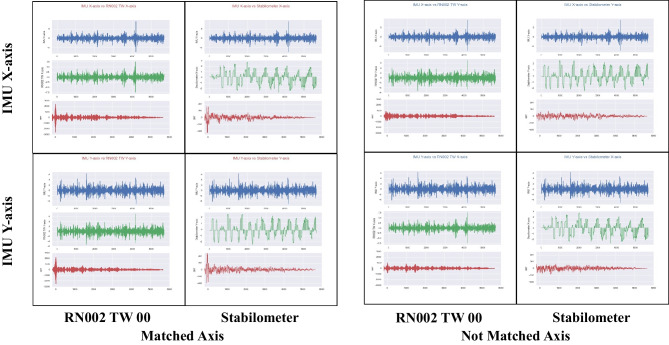
Examination of data by calculating cross-correlation functions. In each figure, the first row represents the displacement data in the IMU, the second row represents the displacement data from the stabilometer or RN002 TW, and the third row represents the cross-correlation function of the data. If the axes are matched, the cross-correlation function signal was observed near the start of the recording and then decayed. If the axes are not matched, the signal was not clear, and noise was high over time

To verify whether the signal due to the load on the stabilometer was correctly detected by the RN002 TW device, a cross-correlation function was created to assess its correlation with axis displacement data of the stabilometer, with respect to the IMU. An example of this calculation is shown in Fig. 3. When the direction of the XY axis of the reference IMU and each device matched, the value of the cross-correlation function was large only at early timepoints of each experiment (within 1 s), and the value of the correlation function became small afterward (the cross-correlation function had such a property because input signals were random). However, when axial directions were not coincident, values of the cross-correlation function remained small throughout, indicating that equivalent measurements were successfully performed by each device.

#### Calculation of evaluation values for each device

In each trial, displacement data for each device, with the analysis start point aligned with the trigger signal, were cut out until the signal ended. Total trajectory length, outer peripheral area, and root mean square (RMS) area drawn by each device were calculated. In the formulas below, n is the number of displacement data points cut out in each trial.

#### The total trajectory length

The total trajectory length was considered the total distance a trajectory point moved throughout data collection. It was calculated as follows (${{\varvec{x}}}_{{\varvec{i}}}$: ${\varvec{i}}$ -th displacement data x-coordinate, ${{\varvec{y}}}_{{\varvec{i}}}$: ${\varvec{i}}$ -th displacement data y-coordinate):

$$total\;length=\sum\limits_{i=1}^{n-1}\left(\sqrt{{{(x}_{i+1}-x_i)}^2+({y_{i+1}-y_i)}^2}\right).$$

#### Outer peripheral area

Outer peripheral area refers to the area enclosed within a trace of the outermost part of a locus (i.e., the outline of the locus), and was calculated as follows:

$$outer\;peripheal\;area=\sum\nolimits_{j=1}^{360}S_{j.}$$

The area around the origin is divided into 360 equal parts (1° each), and each point is classified within 360 regions. The point in each of the divided regions that is the farthest from the origin (defined as point $P$) must be identified, and $r_j$ was defined as the distance between that point and the origin. Next, the area (defined as $S_j$) of the triangle connecting point $P$, the origin of gravity center motion, and adjacent regions were determined, and all were integrated using the following equation:

$$S_j=\sum\nolimits_{j=1}^{360}\frac{r_jr_{j+1}\mathbf{sin}\theta}2(r_{361}=r_1,\theta=1^\circ).$$

#### RMS area

The RMS area is the area of a circle whose radius is the RMS value in XY 2 dimensions. RMS was calculated as follows (The average value in each direction is defined as the locus origin $\lbrack x_{mean}$**,** $y_{mean}$]):

$$x_{mean}=\sum\nolimits_{i=1}^nx_i,$$

$$y_{mean}=\sum\nolimits_{i=1}^ny_i.$$

The RMS value in XY 2 dimensions was obtained using the following formula:

$$root\;mean\;square\;(RMS)=\sqrt{\frac1n\sum\nolimits_{i=1}^n\left({{(x}_i-x_{mean})}^2+\Delta({y_i-y_{mean})}^2\right)}.$$

Effective area was calculated using the following formula:

$$RMS\;AREA=\pi{(RMS)}^2$$

### Statistical analysis

All statistical analyses were performed using Python, version 3.9.7. To evaluate the correlations between each device for the total trajectory length, outer peripheral area, and RMS area, Spearman’s correlation coefficients were calculated. The false discovery rate (FDR; Q < 0.05) method was used to correct multiple comparisons. Statistical significance was set at P < 0.05. The comparability and agreement levels with IMU, Stabilometer, and RN002 TW in Total trajectory length, outer peripheral area, and RMS area were calculated using the Bland–Altman method.

## Results

**Fig. 4:**
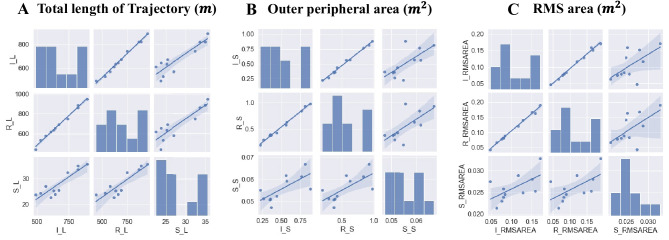
Correlations between the IMU, RN002 TW, and Stabilometer. **A** Total length of Trajectory. I_L – length of IMU. R_L – length of RN002 TW. S_L – length of Stabilometer. **B** Outer peripheral area. I_S – area of IMU. R_S – area of RN002 TW. S_S – area of Stabilometer. **C** RMS area. I_RMSAREA – area of IMU. R_RMSAREA – area of RN002 TW. S_RMSAREA – area of Stabilometer. The straight line in the graphs represents the regression between the variables. A significant correlation between the total trajectory length and outer peripheral area of head displacement measured via a wearable device and the center-of-gravity movement measured via stabilometry

Total trajectory length determined via IMU and RN002 TW was correlated (IMU–RN002 TW [r = 1.00, p–FDR < 0.001]; RN002 TW–stabilometry [r = 0.818 (0.429–0.951), p–FDR = 0.004], and stabilometry–IMU [r = 0.818 (0.429–0.951), p–FDR = 0.002]). The RMS area showed a positive correlation between IMU–RN002 TW (r = 0.982 (0.929–0.995), p–FDR < 0.001), but no significant correlation was found between RN002 TW–Stabilometry and Stabilometry–IMU. A positive correlation between outer peripheral IMU–RN002 TW (r = 0.973 (0.895–0.993), p -FDR < 0.001), RN002 TW–Stabilometry (r = 0.691 (0.155–0.913), p–FDR = 0.026), and stabilometry (r = 0.664 (0.106–0.904), p–FDR = 0.026) values were observed (Fig. 4, Table 1).

**Table 1:** The relationship between the IMU, RN002 TW 00, and Stabilometer

	r	r 95%CI	p	p*
**Total length of Trajectory**				
IMU–RN002 TW	1.000		< .001	< .001
RN002 TW–Stabilometry	0.818	(0.429–0.951)	0.002	0.002
Stabilometry–IMU	0.818	(0.429–0.951)	0.002	0.002
**Outer peripheral area**				
IMU–RN002 TW	0.973	(0.895–0.993)	< .001	< .001
RN002 TW–Stabilometry	0.691	(0.155–0.913)	0.019	0.026
Stabilometry–IMU	0.664	(0.106–0.904)	0.026	0.026
**Root mean square area**				
IMU–RN002 TW	0.982	(0.929–0.995)	< .001	< .001
RN002 TW–Stabilometry	0.600	(0–0.882)	0.051	0.077
Stabilometry–IMU	0.536	(–0.094–0.860)	0.089	0.089

a CI; confidence interval b p*; P-value after FDR adjustment c P < 0.05 was considered significant

**Fig. 5:**
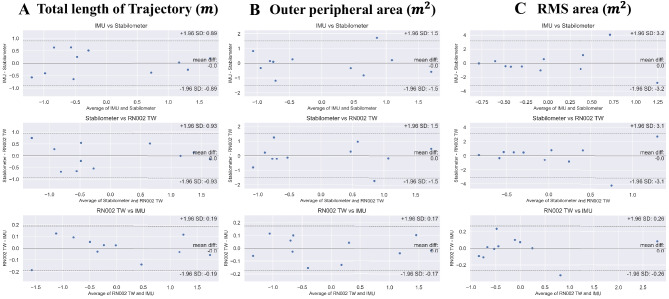
Bland–Altman plots along with the mean error and the 95% limits of agreement (CI95%) for comparison between IMU, RN002 TW, and Stabilometer readings. **A** Total length of Trajectory. **B** Outer peripheral area. **C** RMS area

The Bland–Altman analysis was conducted to compare between IMU data and Stabilometer data (Fig. 5A), between Stabilometer data and RN002 TW data (Fig. 5B), and between RN 002 data and IMU data (Fig. 5C) with standardization. Almost all IMU data, Stabilometer data and RN002 TW data were distributed within 1.96 SD. In terms of Total length of Trajectory, the limits of agreement between IMU data and Stabilometer data ranged from –0.89 to 0.89. The limits of agreement between Stabilometer data and RN002 TW data ranged from –0.93 to 0.93. The limits of agreement between RN 002 TW data and IMU data ranged from –0.19 to 0.19. In terms of the outer peripheral area, the limits of agreement between IMU data and Stabilometer data ranged from –1.5 to 1.5. The limits of agreement between Stabilometer data and RN002 TW data ranged from –1.5 to 1.5. The limits of agreement between RN 002 TW data and IMU data ranged from –0.17 to 0.17. In terms of RMS area, the limits of agreement between IMU data and Stabilometer data ranged from –3.2 to 3.2. The limits of agreement between Stabilometer data and RN002 TW data ranged from –3.1 to 3.1. The limits of agreement between RN 002 TW data and IMU data ranged from –0.26 to 0.26.

## Discussion

Studies of body sway using 3D accelerometers and stabilometry have been conducted in the past. Further, the equivalence of body sway between stabilometry and motion capture video has been reported using direct kinematic measures of postural sway obtained from motion capture [14]. Utilizing the characteristics of devices that were previously demonstrated, we examined whether wearable devices are potentially clinically useful by comparing data obtained via wearable devices with that of a stabilometer currently used in medical practice.

In general, center of gravity and body sway are not the same index. Uprightness is considered a special type of motion because the center of mass (COM) moves within a specific range when humans maintain an upright posture [15]. In addition, since the control mechanism of the center of mass of the body is associated with external forces and voluntary movements in normal subjects that involve a complex interplay between the recovery response and voluntary movements [16], the range (area) and speed of COM motion (velocity) are related the capacity of an individual to maintain an upright posture, and are therefore ideal indicators of the equilibrium function. However, because determining COM values during spontaneous standing requires a complex calculation [17], the COP, which can be easily determined, has been used instead of the COM to evaluate equilibrium dysfunction. Previous studies have shown a correlation between stabilometry and body sway during data during stationary standing [18].

As indicated in the results, a significant correlation between the total trajectory length and outer peripheral area of head displacement measured via a wearable device and the center-of-gravity movement measured via stabilometry. In similar studies assessing the use of wearable devices, RMS calculated using an acceleration signal was the most frequently used outcome measure [19]. In the present study, although no significant RMS area difference between that measured via a wearable device and the center-of-gravity movement measured via stabilometry was observed, a positive correlation was expected. The high degree of correlation between each parameter evaluated via IMU and RN002 TW indicates that the RN002 TW is comparable to the reference IMU in terms of performance. This indicates that the performance and accuracy of the RN002 TW are the same as those of the IMU.

Generally, stabilometry is used to track symptoms of vertigo (equilibrium dysfunction) [20, 21]. The number of patients complaining of vertigo continues to increase, with the prevalence of dizziness in patients over 60 years of age being 30% [22]. One cause of vertigo is inner ear dysfunction, as exemplified by Meniere's disease, which has a recently reported prevalence of 0.51%, a value much higher than previously estimated [23]. Symptoms of Meniere’s disease are often variable and recurrent. During medical examinations, it is important to accurately assess the occurrence of a patient's dizziness symptoms over time. However, because tests must be performed at medical institutions, the only results recorded are from visits, symptoms experienced at any time other than a doctor’s evaluation must be considered based only on subjective patient complaints. This makes it difficult for medical staff to objectively assess the degree, variability, and transition of dizziness symptoms [24], which hinders the ability of medical staff to properly understand dizziness symptoms, making their treatment difficult. Quantitative assessments that track fluctuating symptoms of inner ear dysfunction are still under study [25].

These results indicate that wearable devices, which are widely available on the market, have the potential to be used for out-of-hospital gravity sway testing after the addition of appropriate signal processing software. It is important to be able to objectively track symptoms of dizziness over time from outside the hospital in a way that is comparable to that of a medical examination. Although head motion and center-of-gravity sway do not result in the same type of motion, head motion tracking may be useful for measuring equilibrium outside the hospital.

This study had some limitations. First, the study did not involve human subjects, and instead employed a simplified model of the human body, meaning that experiments were not conducted on actual healthy subjects or patients with balance dysfunction. As previously discussed, there is a complex interplay between righting reflex and voluntary movement elements in actual subjects, and these control mechanisms may require consideration. The inverted pendulum model is sometimes applied to compensate for this gap [16, 26], whereas a correlation close to 1 has been predicted between trunk acceleration and COP displacement that occurs when the body follows a path predicted via the inverted pendulum model [27]. Indeed, a high degree of correlation has been observed between the COP and center-of-gravity amplitude during standing [28]. We believe that data acquired from equilibrium function tests using wearable devices have the potential to correlate highly with conventional results of clinical examinations [19].

## Conclusions

By comparing the wearable device with the stabilometer currently used in the medical field, the possibility that the device may be used in a clinical setting was assessed. A significant correlation was found between the total trajectory length and outer peripheral area of displacement measured using the wearable device, which assessed head movement, and displacement measured using stabilometry, which assessed center-of-gravity movements. This finding indicated that the device may potentially be useful for performing center-of-gravity movement testing outside the hospital. Future studies will be needed to investigate the possibility of using wearable devices to assess the condition of patients with equilibrium dysfunction. Integrating biopotential signals other than the gravitational sway test (e.g., vestibulo-ocular reflex) with the system in this study may investigate the possibility of using a wearable device to assess the condition of patients with equilibrium dysfunction outside the hospital.

## Data Availability

Data are available upon a proper request by contacting Dr. Masato Fujioka: mtfuji@kitasato-u.ac.jp.

## Abbreviations

COM: Center of mass
COP: Center of pressure
DC: Direct current
FDR: False discovery rate
HCI: Human Computer Interaction
HPFs: High pass filters
IMU: Inertial measurement unit
RMS: Root mean square
